# A Hierarchical Niche Structure, Not Niche Partitioning, Organizes a Seasonally Dynamic Flea Community

**DOI:** 10.1002/ece3.72951

**Published:** 2026-01-12

**Authors:** Rui Geng, Yakun Liu, Guokang Chen, Haizhou Yang, Shuai Yuan, Heping Fu

**Affiliations:** ^1^ College of Grassland Science Inner Mongolia Agricultural University Hohhot China; ^2^ Key Laboratory of Grassland Rodent Ecology Rodent Pest Control at Universities of Inner Mongolia Autonomous Region Hohhot China; ^3^ Key Laboratory of Grassland Resources Ministry of Education P.R. of China Hohhot China

**Keywords:** community hierarchy, functional groups, host–parasite network, multidimensional niche, niche breadth, parasite community assembly

## Abstract

Interactions in single‐host–parasite systems provide a tractable framework for understanding the ecological mechanisms that maintain community stability; yet, the link between species' multidimensional niches and their functional roles within these networks remains underexplored. Here, we integrated network topology, multidimensional niche analysis, and functional group delineation to investigate the adaptive strategies and assembly rules of a 12‐species flea community on Mongolian gerbils ( *Meriones unguiculatus* ). The host‐flea network was characterized by a stable, nested structure and exhibited strong seasonal dynamics, with connectivity peaking in summer and modularity increasing in autumn. To understand the underlying mechanisms, we quantified the niche breadth of each species along four identified ecological gradients. Our analysis revealed that the community was organized along a steep hierarchy of generalization. Two hyper‐generalist species (*Nosopsyllus laeviceps kuzenkoui* and *Xenopsylla conformis conformis*), characterized by near‐maximal niche breadth and core network positions, dominated the community. A broad niche was a major determinant of a species' role, showing a significant positive association with a wider range of exploited hosts (Wilcoxon test: *p* = 0.03, effect size = 0.82). In contrast, specialist species, such as the extreme specialist (*Ophthalmopsylla jettmari*), were confined to the network's periphery and a narrow subset of ecological conditions. Clustering based on the multidimensional niche profiles identified four distinct functional groups, reflecting a clear hierarchy of ecological strategies. Overall, this study suggests that, within this seasonally dynamic system, a hierarchical niche structure, rather than complex trade‐offs, is a primary organizing principle, providing a more nuanced understanding of stability in parasitic systems.

## Graphical abstract

Using seasonal data from a single‐host system (Mongolian gerbil‐flea), we show that community organization is governed by a hierarchical niche structure rather than classic niche partitioning. Network analyses reveal a persistent generalist core with seasonally shifting peripheries (spring assembly, summer integration, autumn reorganization). A four‐dimension niche framework explains species strategies and separates “super‐generalists” from extreme specialists.

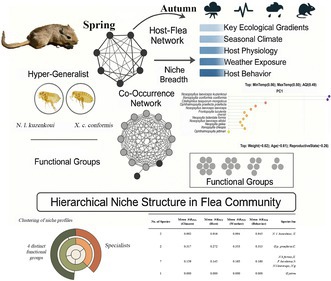

## 1 Introduction

Interactions between parasites and their hosts are fundamental to the maintenance of biodiversity and community structure, and they play an important role in community assembly and species coexistence (Pilosof et al. 2015). With the emergence of network ecology, parasitic interaction networks, typically constructed as bipartite host–parasite matrices, have become a widely used tool in multi‐host–multi‐parasite systems for identifying structural hub species, tracing interaction pathways, and characterizing host‐dependence patterns (Tylianakis et al. 2007; Albery et al. 2020; Runghen et al. 2021). Such studies effectively illuminate community‐level interaction architecture and dynamics. However, in multi‐host systems, parasite occurrence and interaction patterns often reflect a composite of host environmental context, transmission opportunity, and host relatedness, which can obscure parasite‐centered inference about ecological responses and functional differentiation (Dallas and Presley 2014). Comparative syntheses further indicate that parasite species richness varies systematically with host attributes and ecological context across diverse host–parasite systems, highlighting the need to consider multiple, interacting drivers rather than a single axis (Kamiya et al. 2014). Moreover, phylogeographic work on generalist flea species shows that parasite life history can interact with host history and vicariance, such that parasite–host association matters for spatial structure and inferred connectivity (van der Mescht et al. 2015). By comparison, single‐host–multiple‐parasite network analysis offers a more tractable framework: by substantially reducing variation in host identity and associated trait heterogeneity, it enables more focused investigation of how parasites respond to microenvironmental gradients and how functional roles emerge within parasite assemblages (Dallas and Cornelius 2015; Pilosof et al. 2015). Nonetheless, empirical work on single‐host parasite networks remains limited, and detailed studies that explicitly couple network‐structural metrics with parasite niche characteristics are especially scarce. Addressing this gap is essential if we are to gain mechanistic insight into how parasite community structure reflects environmental gradients and host‐individual traits. To gain such mechanistic insight, niche analysis provides a powerful means to quantify the resource‐use differentiation and potential competitive interactions that underpin community structure.

Variability in parasitic network structure is frequently associated with heterogeneous flea responses to environmental conditions and host‐related resources (Bellay et al. 2011; Takemoto et al. 2014; Bellay et al. 2018). Quantitative niche metrics, such as breadth, specialization, and overlap, can describe the extent of resource use, highlight potential ecological competition or functional redundancy, and establish a foundation for assessing species' ecological roles within interaction networks (Haverkost et al. 2010). Yet most existing studies have considered only isolated host traits or environmental drivers, without jointly accounting for host‐environment gradients. This limitation restricts our ability to capture the combined patterns of flea niche breadth, overlap, and differentiation (Soberon and Peterson 2005; Mouillot et al. 2013). To address this gap, we established a host‐flea network using the Mongolian gerbil ( *Meriones unguiculatus* ) as the sole host and its ectoparasitic fleas as study organisms. By minimizing variation in host identity, this system offers a controlled framework for examining flea ecological differentiation and community assembly mechanisms under microenvironmental gradients (Krasnov et al. 2014). To further investigate interspecific relationships and functional differences among fleas, we integrated analyses of network structure, multidimensional niche metrics, and non‐metric multidimensional scaling (NMDS) models. This integrative approach enables a joint evaluation of structural, functional, and niche dimensions, providing new insights into the mechanisms of flea ecological adaptation and offering a tractable pathway toward micro‐scale, function‐oriented parasitic ecology (Beugnet et al. 2009).

Based on this framework, we specifically hypothesized that: (1) flea species with broader, more generalized niche traits would occupy more central and influential positions within the community network and (2) the network's structure, particularly its modularity, would exhibit significant seasonal dynamics corresponding to shifts in environmental gradients and resource availability. Testing these hypotheses will provide direct insight into the functional assembly rules of this parasite community.

## 2 Materials and Methods

### 2.1 Host Capture and Flea Collection

From March to November 2024, seven plots were randomly selected within the main distribution area of *M. unguiculatus* . Each plot covered approximately 1 ha where 100 live traps were placed in a 10 m × 10 m grid with 10 m spacing between traps and rows. Fresh peanuts were used as bait. Considering that *M. unguiculatus* is a typical diurnal rodent, traps were opened from 06:00 to 19:00 and checked every 2–3 h. Captured individuals were promptly removed, placed into separate cloth bags, and transported to the field station laboratory for flea collection. Trapping was conducted continuously for 15–20 days per month. In total, 929 *M. unguiculatus* were captured (♀: 493, ♂: 436), and 5293 fleas were collected.

In the laboratory, gerbils were anesthetized using isoflurane (Lang 1996), and fleas were collected following standard protocols. The cloth bags were also checked to avoid missing any fleas. All fleas were immediately preserved in 95% ethanol‐filled cryotubes for subsequent morphological identification. Host characteristics, including sex, age, body weight, and reproductive status, were recorded. After processing, the gerbils were released at the capture site. All animal handling procedures complied with the guidelines of the Ethics Committee of Inner Mongolia Agricultural University (no. NND2025007).

### 2.2 Network Construction and Visualization

To examine the interaction structure between hosts and fleas, we constructed three types of networks: a host‐flea bipartite network, a flea co‐occurrence network, and a seasonal dynamic network. To ensure data reliability, only host samples infected with fleas were included. The host‐flea bipartite network was constructed from an adjacency matrix, and subnetworks were generated by retaining nodes with degree ≥ 3. Layouts were optimized using the Fruchterman–Reingold force‐directed algorithm, with node size scaled by degree and node color distinguishing hosts from fleas. Network topology was evaluated using standard metrics, including connectance, nestedness, and modularity, with modularity optimized using Beckett's algorithm (Dormann et al. 2008).

The flea co‐occurrence network was derived from the host–parasite abundance matrix by calculating pairwise Bray–Curtis similarities. A threshold (> 0.1) was applied to remove weak associations, retaining only significant co‐occurrence relationships. In the resulting weighted undirected network, nodes represented flea species, and edge weights indicated the strength of co‐occurrence. Network communities were identified using the Louvain algorithm (Blondel et al. 2008).

### 2.3 Identification of Major Ecological Gradients via Principal Component Analysis

To reduce the dimensionality of the host and environmental variables and identify the primary independent axes of ecological variation, we performed a Principal Component Analysis (PCA). The input dataset comprised 11 variables (MaxTemp, MinTemp, WindSpeed, WindDirection, Precipitation, AQI, Sex, Weight, CaptureStatus, Age, ReproductiveState) for each flea‐infected host.

The analysis was conducted using the prcomp function from the stats package in R (version 4.4.2). Prior to analysis, all variables were centered and scaled to unit variance (scale. = TRUE) to eliminate the influence of different measurement units. We determined the number of components to retain for subsequent analysis using the widely accepted Kaiser–Guttman criterion, which retains all components with eigenvalues greater than 1. This resulted in the selection of the first four principal components (PC1–PC4), which collectively explained 60.49% of the total variance in the host‐environment dataset. The scores of these four PCs for each host were then used as proxies for the host's position along four distinct ecological gradients.

### 2.4 Quantification of Flea Niche Indices

To assess flea niche breadth along each of the four identified ecological gradients, we developed a multidimensional niche breadth index. The calculation was performed programmatically in R for each of the 12 flea species along each of the four principal component axes.

First, for each principal component *k* (where *k* = 1, 2, 3, 4), the absolute value of the component score for each host *i*, denoted as $w_{\mathrm{ik}}=\left|{\mathrm{PC}}_{\mathrm{ik}}\right|$, was used as a weight. This weight represents the host's relative position along that specific ecological gradient. Using this weight, we calculated a weighted abundance vector, $P_{\mathrm{jk}}^{*}$, for each flea species *j* along each gradient *k*. The elements of this vector, $p_{\mathrm{ijk}}^{*}$, representing the weighted abundance on host *i*, were defined as:

(1) $$p_{\mathrm{ijk}}^{*}=w_{\mathrm{ik}}\times x_{\mathrm{ij}}$$

where $x_{\mathrm{ij}}$ is the abundance of flea species *j* on host *i*.

Subsequently, we calculated two metrics based on the weighted abundance vector $P_{\mathrm{jk}}^{*}$: the standardized Levins' index $B_{\mathrm{std}}$, a measure of the evenness of resource use, and the Shannon diversity index (*H*), which incorporates both richness and evenness. To avoid errors with zero values, a small constant (1e‐6) was added to all abundances prior to normalization within the functions. The formulas are as follows:

(2) $$B_{\mathrm{jk}}=\frac{1}{\sum_{i=1}^{N}{\left(\frac{p_{\mathrm{ijk}}^{*}}{\sum_{i=1}^{N}p_{\mathrm{ijk}}^{*}}\right)}^{2}}\;\text{and}\;B_{\mathrm{jk},\mathrm{std}}=\frac{B_{\mathrm{jk}}-1}{N-1}$$

(3) $$H_{\mathrm{jk}}=-\sum_{i=1}^{N}\left(\frac{p_{\mathrm{ijk}}^{*}}{\sum_{i=1}^{N}p_{\mathrm{ijk}}^{*}}\right)\ln\left(\frac{p_{\mathrm{ijk}}^{*}}{\sum_{i=1}^{N}p_{\mathrm{ijk}}^{*}}\right)$$

where *N* is the total number of infected hosts.

To create a robust, composite measure of niche breadth, the resulting $B_{\mathrm{jk},\mathrm{std}}$ and $H_{\mathrm{jk}}$ values were first standardized within each PC axis *k* using *Z*‐scores:

(4) $$Z_{\mathrm{jk},B}=\frac{B_{\mathrm{jk},\mathrm{std}}-\mu_{B_{k}}}{\sigma_{B_{k}}}\;\text{and}\;Z_{\mathrm{jk},H}=\frac{H_{\mathrm{jk}}-\mu_{H_{k}}}{\sigma_{H_{k}}}$$

where μ and σ are the mean and standard deviation of the respective indices across all species for a given PC axis *k*.

The two *Z*‐scores were then averaged to form a single composite niche breadth index ($NB_{\mathrm{jk}}$) for each species *j* along each gradient *k*:

(5) $$NB_{\mathrm{jk},\text{comp}}=\frac{Z_{\mathrm{jk},B}+Z_{\mathrm{jk},H}}{2}$$

Finally, to facilitate comparison, this composite index was normalized to a 0–1 scale using Min‐Max scaling, resulting in the final multidimensional niche breadth index,

$NB_{\mathrm{jk}}$:

(6) $$NB_{\mathrm{jk}}=\frac{NB_{\mathrm{jk},\text{comp}}-\min\left(NB_{k,\text{comp}}\right)}{\max\left(NB_{k,\text{comp}}\right)-\min\left(NB_{k,\text{comp}}\right)}$$

This entire process resulted in four distinct, standardized niche breadth indices ($NB_{\mathrm{PC}1},NB_{\mathrm{PC}2}$, $NB_{\mathrm{PC}3}$, $NB_{\mathrm{PC}4}$) for each flea species. To evaluate the uncertainty of these estimates, we performed a bootstrap resampling procedure (500 iterations) using the boot package to calculate the 95% percentile confidence intervals (CIs) for the final $NB_{\mathrm{jk}}$ indices.

In this study, we use hyper‐generalist to refer to flea taxa combining near‐maximal standardized multidimensional niche breadth (*NB* close to 1 across the four gradients) with core positions in the co‐occurrence network (high connectivity/degree), whereas extreme specialist refers to taxa with near‐minimal niche breadth (*NB* approaching 0) and consistently peripheral network positions (low connectivity). These terms are used as metric‐based descriptors of niche breadth and network position rather than as claims about evolutionary mechanism. Accordingly, the term steep hierarchy of generalization describes the resulting community structure characterized by a sharp discontinuity in niche breadths, where a small number of hyper‐generalists coexist with a majority of restricted specialists, rather than a continuous gradient of generalization.

### 2.5 Environmental Gradient Response Analysis

**FIGURE 1:**
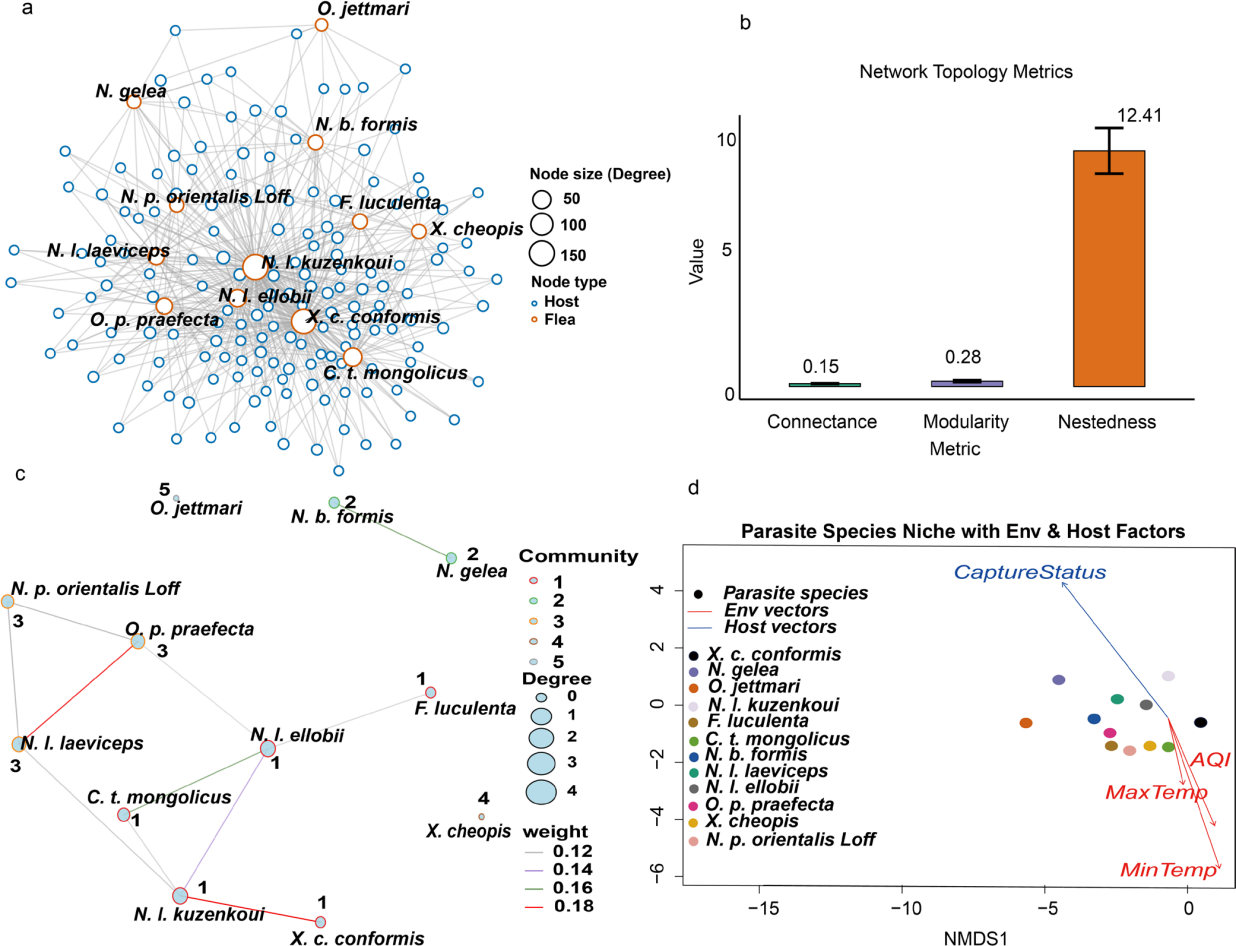
(a) Overall bipartite network structure between Mongolian gerbils and their fleas; (b) topological metrics of the network; (c) flea co‐occurrence network and community structure; (d) Non‐metric multidimensional scaling (NMDS) ordination of flea species based on Bray–Curtis dissimilarities. Environmental and host variables were fitted to the ordination using permutation tests (999 permutations); arrows show the direction and relative strength of significant variables (*p* < 0.05). Generalist species are positioned near the ordination center, whereas specialists cluster toward the margins along specific environmental‐host gradients.

NMDS was applied to assess variations in flea community composition. A dissimilarity matrix was constructed using Bray–Curtis distances, with a two‐dimensional solution space (*k* = 2). To ensure stability, multiple random initializations were performed. Environmental variables (MaxTemp, MinTemp, WindSpeed, WindDirection, Precipitation, AQI) and host traits (Sex, Weight, CaptureStatus, Age, ReproductiveState) were fitted to the NMDS ordination using permutation tests (999 iterations), and significant factors were selected at *p* < 0.05. In the ordination plots, different flea species were distinguished by colors, while environmental and host variables were represented by arrow vectors indicating their direction and strength (Figure 1d). All analyses were conducted in R using the vegan package.

### 2.6 Statistical Analysis

#### 2.6.1 Linking Ecological Traits to Multidimensional Niche Breadth

To investigate whether niche breadth in different ecological dimensions corresponds to differences in fundamental ecological traits, we performed four independent sets of analyses. For each of the four niche breadth indices ($NB_{\mathrm{PC}1}$ to $NB_{\mathrm{PC}4}$), the 12 flea species were categorized as “generalists” (upper quartile) or “specialists” (lower quartile). Species in the middle range were excluded to enhance contrast. For each categorization, differences in five key ecological traits (host richness, total abundance, habitat richness, seasonal richness, and mean parasitic intensity) between the two groups were evaluated using the non‐parametric Wilcoxon rank‐sum test. Wilcoxon effect sizes (*r*) were calculated to assess the magnitude of group differences.

#### 2.6.2 Identification of Functional Groups

To identify functional groups based on the species' overall multidimensional niche strategies, we performed k‐means clustering. The analysis was based on the four standardized and normalized niche breadth indices ($NB_{\mathrm{PC}1},NB_{\mathrm{PC}2}$,$NB_{\mathrm{PC}3}$, $NB_{\mathrm{PC}4}$) for the 12 flea species. This approach clusters species with similar combinations of niche breadths across the four ecological dimensions, thus defining functional groups based on their complete ecological strategy profile.

All statistical analyses and visualizations were performed in R (version 4.4.2), with the rstatix package used for hypothesis testing and effect size estimation, and ggpubr employed for graphical visualization.

## 3 Results

### 3.1 Flea Community Composition and Overall Structure

A total of 5293 fleas, representing 12 species from 11 genera and 6 families, were collected from 929 Mongolian gerbils. An initial analysis of the overall host‐flea interaction network revealed a highly structured, non‐random community, characterized by low connectance (0.15), high nestedness (12.41, *p* < 0.001), and significant modularity (0.28, *p* < 0.001) (Figure 1a,b).

To specifically investigate interspecific association patterns, we analyzed the flea co‐occurrence network (Figure 1c). This network, comprising 12 species (nodes) and 11 significant co‐occurrence links (edges), was also significantly modular (Modularity = 0.414, *p* < 0.001), with the Louvain algorithm identifying five distinct communities. Crucially, the network exhibited a pronounced core‐periphery structure. Species such as *Nosopsyllus laeviceps kuzenkoui* and *Nosopsyllus laeviceps laeviceps* occupied the network's core, defined by their high connectivity, while species like *Ophthalmopsylla jettmari* and *Xenopsylla cheopis* were positioned at the periphery.

A NMDS ordination further confirmed that flea assemblages were non‐randomly structured across hosts, yielding a robust two‐dimensional solution with a low stress value of 0.12 (Figure 1d). A permutation‐based test revealed that this structure was significantly correlated with both environmental and host‐related variables. Specifically, minimum temperature (*r* ^2^ = 0.077, *p* = 0.001), air quality index (AQI; *r* ^2^ = 0.041, *p* = 0.001), maximum temperature (*r* ^2^ = 0.014, *p* = 0.005), and host capture status (*r* ^2^ = 0.022, *p* = 0.001) showed the strongest and most significant associations with the ordination axes.

Collectively, these initial findings of a well‐defined community structure, driven by a mix of climatic and host‐related factors, motivated a deeper investigation into the specific ecological gradients that shape the functional niches of these flea species.

### 3.2 Seasonal Dynamics of the Mongolian Gerbil‐Flea Interaction Network

**FIGURE 2:**
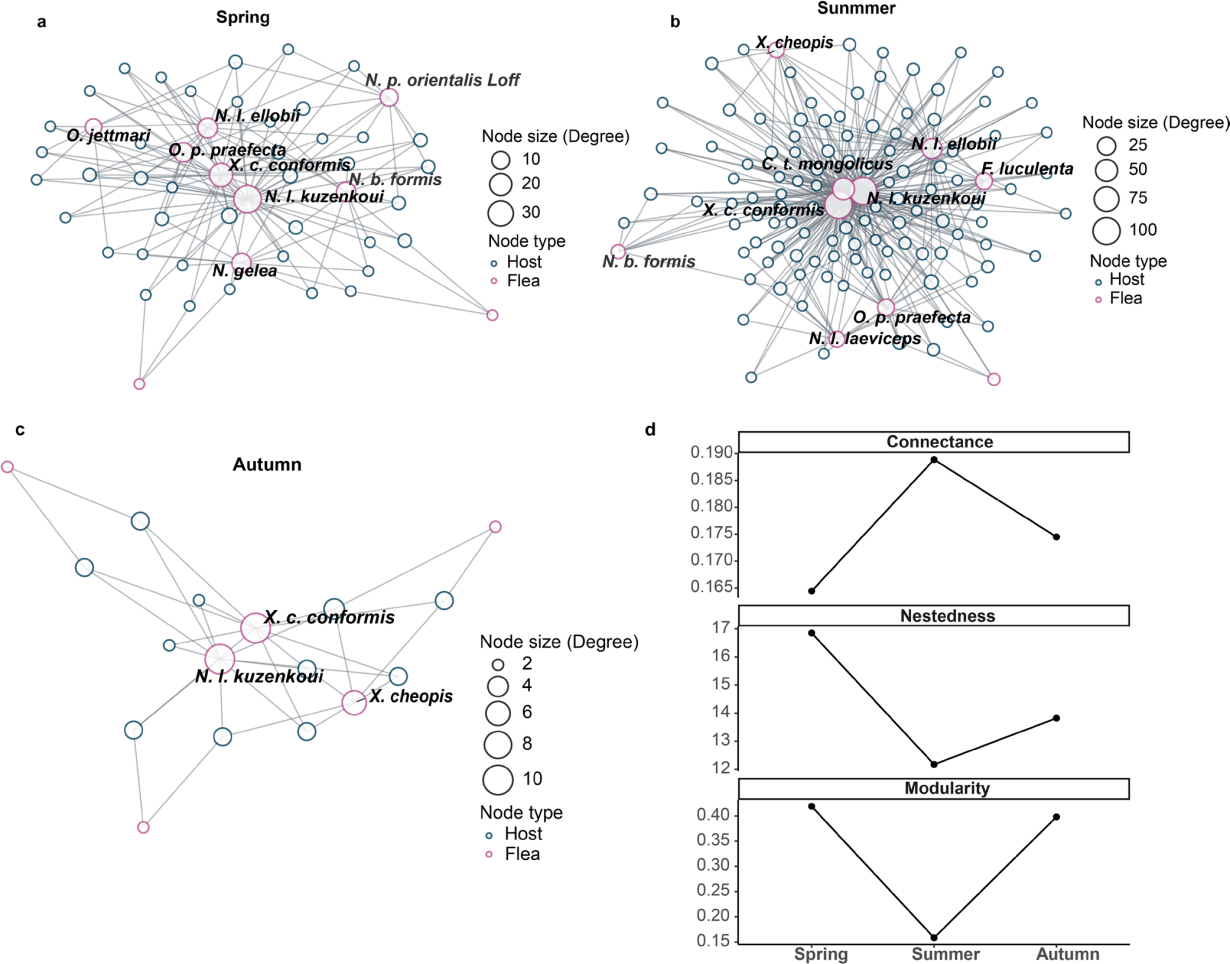
(a–c) The host‐flea interaction networks of Mongolian gerbils across three seasons, while (d) illustrates the seasonal dynamics of three key network metrics; Nestedness is reported as NODF (0–100).

To assess how flea community structure responds to seasonal variation, we constructed host‐flea networks for spring, summer, and autumn (Figure 2a–c) and compared seasonal changes in three key network metrics: connectance, nestedness, and modularity (Figure 2d). The results revealed a distinct sequential pattern of spring assembly, summer expansion, autumn contraction, and restructuring (Figure 2d), indicating that flea communities can rapidly adjust to fluctuations in host activity and environmental conditions.

In spring (Figure 2a), the network included eight flea species, with *N. l. kuzenkoui* exhibiting the highest node degree (~30). Connectance was approximately 0.164, nestedness 16.853, and modularity 0.419, reflecting an incipiently aggregated yet relatively balanced configuration. In summer (Figure 2b), the number of host nodes increased nearly threefold compared with spring, and flea richness expanded to 10 species. Among them, *Xenopsylla conformis conformis*, *Citellophilus tesquorum mongolicus*, and *N. l. kuzenkoui* had node degrees exceeding 75. Network connectance peaked at 0.189, nestedness was 12.77, while modularity declined to 0.158, forming a highly interconnected structure characterized by intensified species interactions and greater network integration. By autumn (Figure 2c), the network contracted sharply, with only three flea species remaining. Both host node numbers and maximum node degree dropped considerably, connectance was 0.174, nestedness 13.821, while modularity increased to 0.389. This shift produced a core‐periphery reorganization, with core species maintaining stable associations and peripheral species largely disappearing.

Taken together, the seasonal dynamics of the three network metrics (Figure 2d) aligned with the structural reorganization observed across seasons. The flea community followed a sequential ecological trajectory of spring assembly, summer expansion and integration, and autumn contraction and restructuring, underscoring its adaptive capacity to host activity and environmental pressures.

### 3.3 The Multidimensional Ecological Space of the Host‐Flea System

**FIGURE 3:**
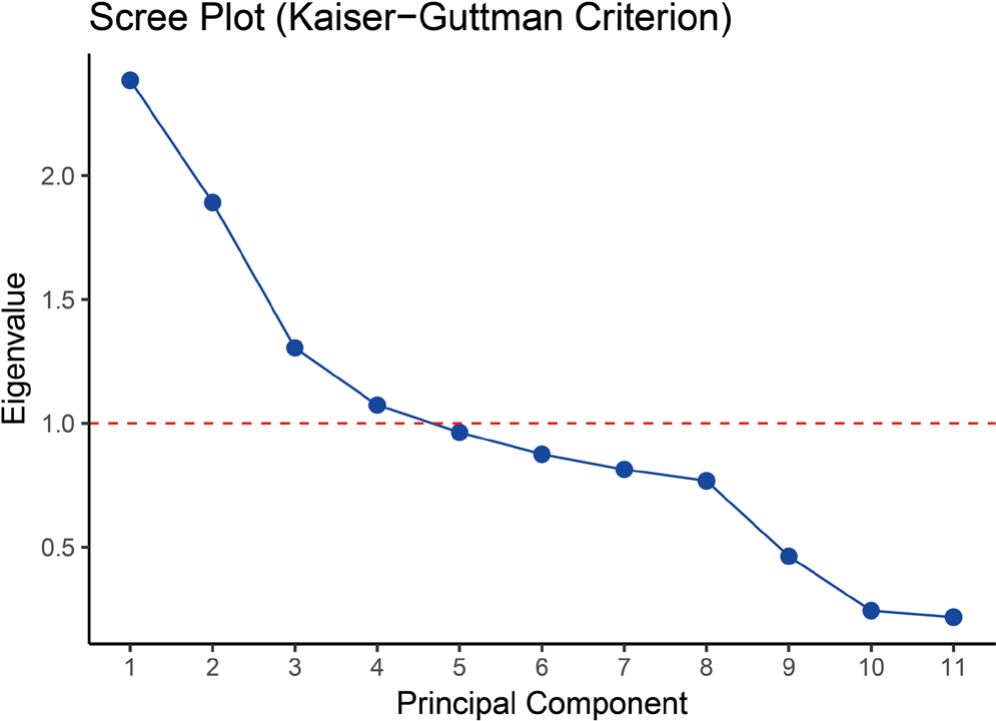
Scree plot of principal component analysis (PCA). Eigenvalues of the first 11 principal components are shown. The red dashed line indicates the Kaiser–Guttman criterion (eigenvalue = 1). Principal components above this threshold (PC1–PC4) were retained for interpretation and subsequent analyses.

Principal Component Analysis (PCA) of the 11 host and environmental variables successfully identified four significant and independent ecological gradients, represented by the first four principal components (PCs). These components, selected based on the Kaiser–Guttman criterion (eigenvalues > 1, Figure 3), collectively explained 60.49% of the total variance in the dataset. By examining the variable loadings, we assigned a distinct ecological interpretation to each axis (Table 1).

**TABLE 1:** PCA variance explained and top contributing variables per principal component.

PC	Eigenvalue	Variance (%)	Cumulative (%)	Top variables (loading; %contrib)
PC1	2.384	21.7	21.7	MinTemp (+0.56; 31.8%); MaxTemp (+0.50; 25.2%); AQI (+0.49; 24.3%); ReproductiveState (+0.24; 5.6%); Age (+0.20; 4.0%)
PC2	1.891	17.2	38.9	Weight (−0.62; 38.8%); Age (−0.61; 37.8%); ReproductiveState (−0.26; 6.7%); Sex (−0.21; 4.3%); MaxTemp (+0.19; 3.6%)
PC3	1.305	11.9	50.7	WindSpeed (+0.57; 32.4%); WindDirection (+0.54; 28.8%); Precipitation (+0.48; 23.5%); CaptureStatus (−0.23; 5.1%); ReproductiveState (−0.19; 3.5%)
PC4	1.073	9.8	60.5	CaptureStatus (+0.73; 53.7%); ReproductiveState (+0.33; 10.9%); WindDirection (+0.33; 10.6%); Sex (+0.27; 7.3%); WindSpeed (+0.26; 6.8%)
PC5	0.963	8.8	69.2	Sex (−0.84; 70.2%); CaptureStatus (+0.31; 9.6%); WindDirection (+0.23; 5.3%); Precipitation (−0.20; 3.9%); Age (+0.18; 3.3%)
PC6	0.875	8	77.2	Precipitation (+0.62; 38.7%); WindDirection (−0.54; 29.5%); CaptureStatus (+0.34; 11.5%); Sex (−0.28; 7.9%); MaxTemp (−0.26; 6.5%)
PC7	0.814	7.4	84.6	WindSpeed (−0.59; 35.0%); ReproductiveState (+0.50; 25.5%); Precipitation (+0.43; 18.3%); WindDirection (+0.40; 15.8%); CaptureStatus (−0.15; 2.4%)
PC8	0.768	7	91.6	ReproductiveState (+0.67; 44.5%); WindSpeed (+0.41; 17.0%); CaptureStatus (−0.39; 15.4%); Precipitation (−0.32; 10.0%); Sex (−0.29; 8.3%)
PC9	0.464	4.2	95.8	AQI (+0.76; 57.7%); MaxTemp (−0.58; 33.8%); WindDirection (+0.17; 3.1%); WindSpeed (+0.15; 2.4%); MinTemp (−0.12; 1.6%)
PC10	0.244	2.2	98	MinTemp (+0.78; 60.4%); MaxTemp (−0.50; 24.7%); AQI (−0.29; 8.3%); WindDirection (+0.20; 4.0%); Precipitation (−0.09; 0.9%)
PC11	0.218	2	100	Age (−0.70; 49.5%); Weight (+0.70; 48.9%); MaxTemp (+0.08; 0.6%); MinTemp (−0.07; 0.5%); AQI (+0.04; 0.2%)

The first principal component (PC1), explaining the largest portion of the variance (21.67%), was strongly and positively correlated with variables exhibiting clear seasonal patterns: minimum temperature (loading = 0.56), maximum temperature (0.50), and the air quality index (0.49). Therefore, PC1 was interpreted as a Seasonal Climate Gradient.

The second component (PC2, 17.19% of variance) was primarily driven by the intrinsic characteristics of the hosts. It showed strong negative loadings from host weight (−0.62) and age (−0.61). Consequently, PC2 was defined as a Host Physiological Status Gradient, effectively distinguishing between older, heavier gerbils (low PC2 scores) and younger, lighter individuals (high PC2 scores).

The third component (PC3, 11.86% of variance) was characterized by high positive loadings from variables related to short‐term weather fluctuations: wind speed (0.57), wind direction (0.54), and precipitation (0.48). This axis was thus interpreted as a Microclimate and Weather Exposure Gradient.

Finally, the fourth component (PC4, 9.76% of variance) was overwhelmingly dominated by a single variable, showing a strong positive loading from capture status (0.73). This axis was therefore interpreted as a Host Behavior and Exposure History Gradient, separating repeatedly captured hosts from those captured for the first time.

Together, these four orthogonal axes defined the multidimensional ecological space within which the niche strategies of the flea species were subsequently evaluated.

### 3.4 Niche Breadth Varies Across Different Ecological Dimensions

**FIGURE 4:**
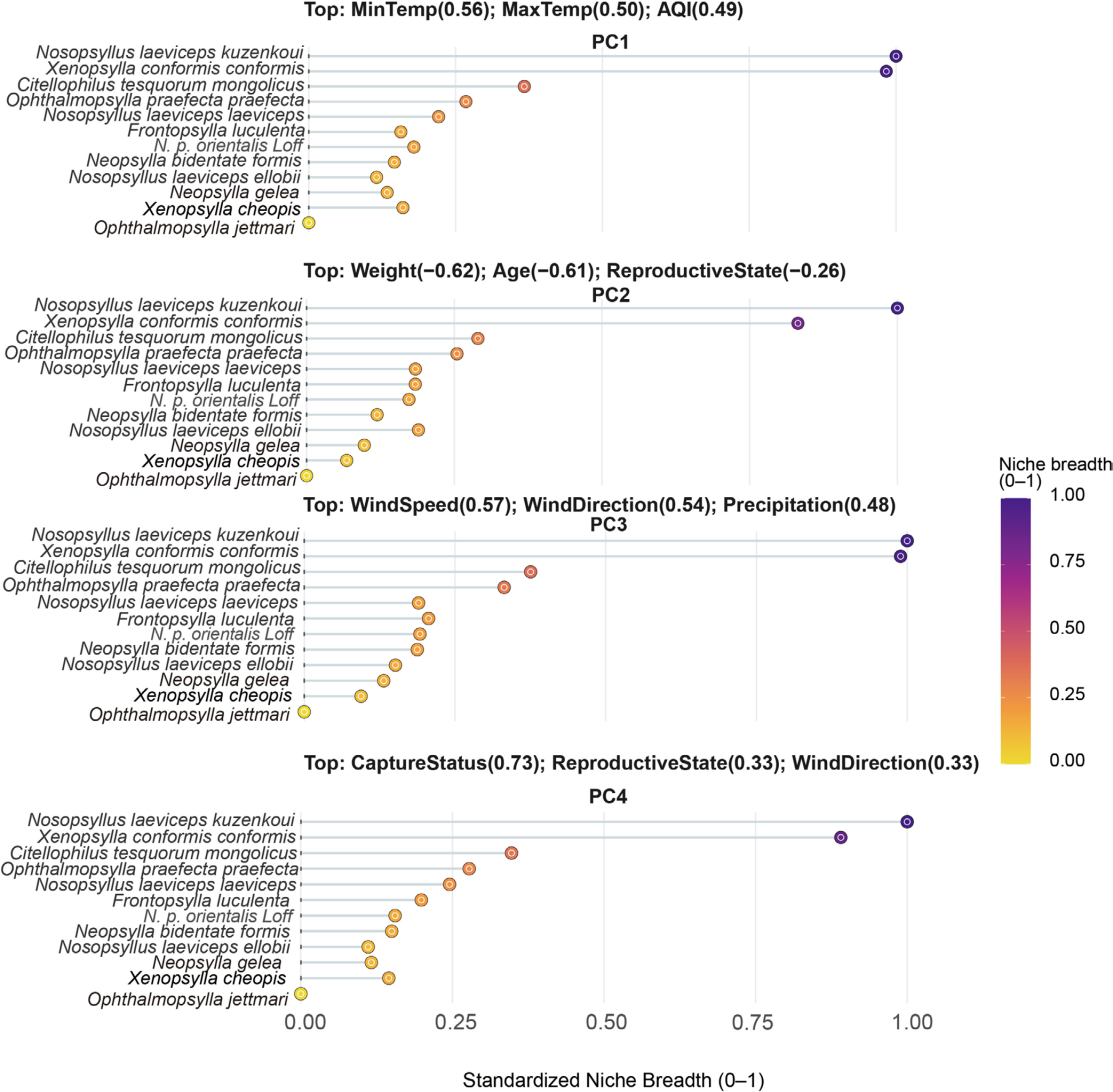
Each panel represents a distinct ecological gradient identified by Principal Component Analysis (PCA): (a) seasonal climate gradient (PC1), (b) host physiological status gradient (PC2), (c) microclimate and weather exposure gradient (PC3), and (d) host behavior and exposure history gradient (PC4). Within each panel, species are ranked along the *y*‐axis. The points represent the standardized niche breadth (*NB*) of each species, calculated as a composite index of the standardized Levins' and Shannon indices and normalized to a 0–1 scale. A value of 1 indicates the broadest possible niche (hyper‐generalist), while a value of 0 indicates the narrowest possible niche (extreme specialist) along that specific gradient. Horizontal error bars represent the 95% bootstrap confidence intervals (Cis) for the niche breadth estimates.

To quantify the functional strategies of each flea species, we calculated their standardized niche breadths along the four previously identified ecological gradients (Figure 4). The results revealed a highly stratified niche structure within the flea community, with a clear hierarchy of generalists and specialists. Most species displayed a consistent niche strategy across all four ecological dimensions rather than extensive trade‐offs.

Two species, *N. l. kuzenkoui* and *X. c. conformis*, unequivocally emerged as the dominant generalists of the community. The species *N. l. kuzenkoui* displayed a maximal niche breadth (*NB* = 1.0) across all four dimensions, indicating an unparalleled ability to thrive under all seasonal climates, on hosts of any physiological status, during variable weather conditions, and on hosts with different behavioral patterns. Similarly, *X. c. conformis* exhibited a near‐maximal niche breadth across all gradients (*NB* ranging from 0.83 to 0.99), positioning it as a co‐dominant, highly adaptable generalist.

In stark contrast, the species *O. jettmari* was identified as an extreme specialist, with a measured niche breadth of zero across all four ecological dimensions. This indicates a highly constrained resource use, confining this species to a very specific and narrow subset of the available host‐environment combinations.

The remaining nine species, including prominent taxa such as *C. t. mongolicus* and *O. p. praefecta*, occupied an intermediate position, behaving as moderate specialists. Their niche breadth values were consistently low to moderate across all four gradients (typically ranging from 0.1 to 0.4), suggesting that while they are not as restricted as *O. jettmari*, they do not possess the broad adaptability of the two dominant generalists.

Overall, these findings depict a community structured by a steep hierarchy of generalization, from the hyper‐generalist dominants (*N. l. kuzenkoui* and *X. c. conformis*) to the extreme specialist (*O. jettmari*). This pronounced hierarchy, rather than a pattern of complex trade‐offs, appears to be the primary organizing principle of niche space in this flea community.

### 3.5 Functional Consequences of Multidimensional Niche Strategies

**FIGURE 5:**
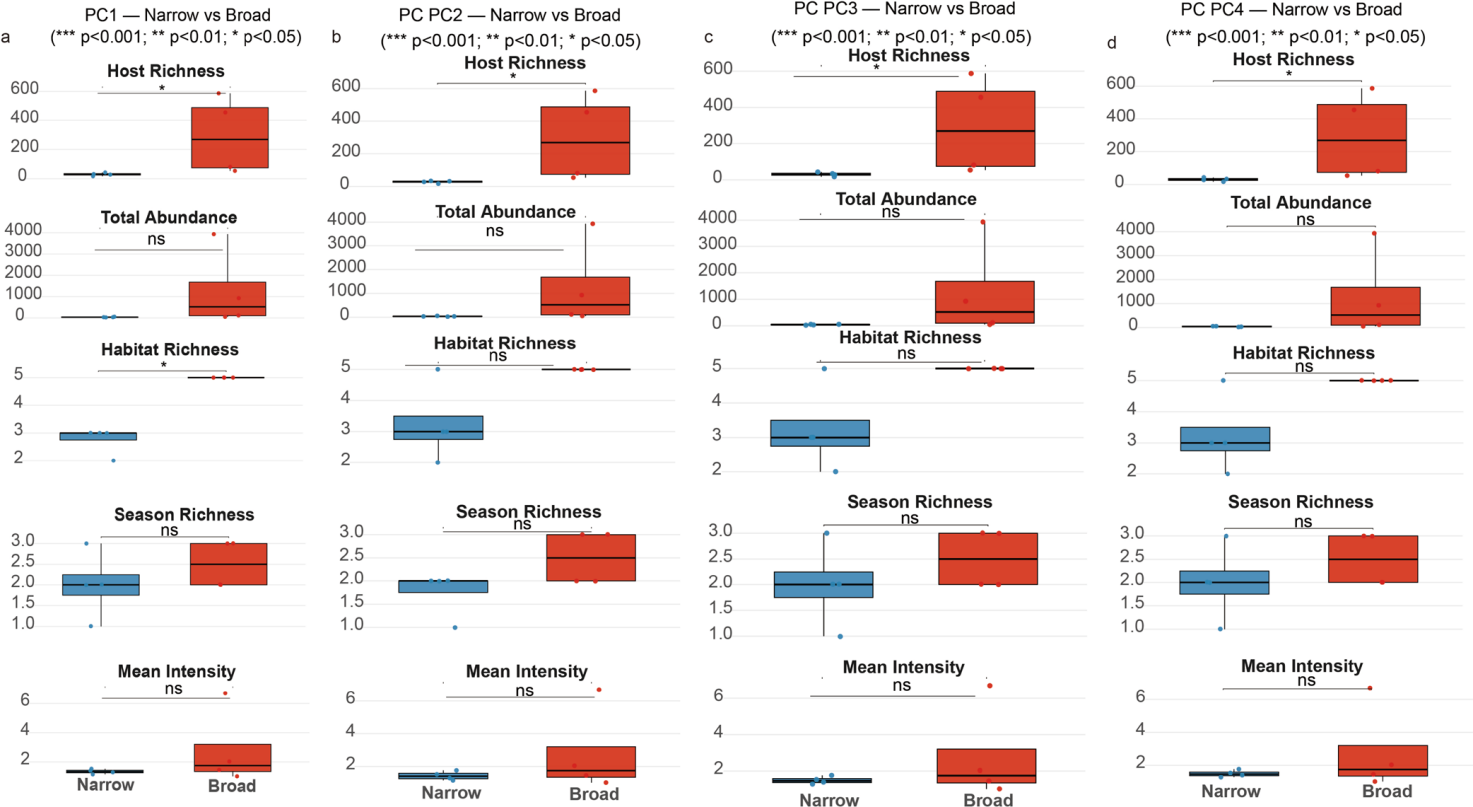
Comparison of ecological traits between generalist and specialist flea species across four niche dimensions. Flea species were categorized as generalists (top 25% niche breadth) and specialists (bottom 25%) based on their niche breadth scores along each of the four ecological gradients separately: (a) seasonal climate gradient (PC1), (b) host physiological status gradient (PC2), (c) microclimate and weather exposure gradient (PC3), and (d) host behavior and exposure history gradient (PC4). Boxplots display the distribution of five key ecological traits for each group: Host Richness, Total Abundance, Habitat Richness, Seasonal Richness, and Mean Intensity. Asterisks (*) indicate a statistically significant difference between the generalist and specialist groups based on a Wilcoxon rank‐sum test (*p* < 0.05). The analysis reveals that generalists consistently utilize a greater richness of hosts across all niche dimensions, while a broader habitat range is specifically associated with generalization on the seasonal climate axis.

To determine whether the observed hierarchy in niche breadth translates into functional differences in the species' ecological roles, we compared five key ecological traits between generalist (upper quartile) and specialist (lower quartile) species, defined separately for each of the four niche dimensions (Table 2, Figure 5).

**TABLE 2:** Statistical comparison of ecological traits between generalist (upper quartile) and specialist (lower quartile) flea species. Species were categorized independently for each of the four niche dimensions (PC1–PC4). Table shows the Wilcoxon rank‐sum test *p*‐values and associated effect sizes (*r*).

PC	Metric	n_narrow	n_broad	*p*	*p_*label	effsize
PC1	HostRichness	4	4	0.0303828219765775	*	0.816496580927726
PC1	TotalAbundance	4	4	0.0606019697120061	ns	0.71443450831176
PC1	HabitatRichness	4	4	0.0177060658073666	*	0.894427190999916
PC1	SeasonRichness	4	4	0.429195300440349	ns	0.335410196624968
PC1	MeanIntensity	4	4	0.47048642205879	ns	0.306186217847897
PC2	HostRichness	4	4	0.0303828219765775	*	0.816496580927726
PC2	TotalAbundance	4	4	0.0606019697120061	ns	0.71443450831176
PC2	HabitatRichness	4	4	0.0667530151696345	ns	0.707106781186547
PC2	SeasonRichness	4	4	0.133614402537716	ns	0.58925565098879
PC2	MeanIntensity	4	4	0.665005542102029	ns	0.204124145231932
PC3	HostRichness	4	4	0.0303828219765775	*	0.816496580927726
PC3	TotalAbundance	4	4	0.110210182673421	ns	0.6160503812913
PC3	HabitatRichness	4	4	0.0667530151696345	ns	0.707106781186547
PC3	SeasonRichness	4	4	0.429195300440349	ns	0.335410196624968
PC3	MeanIntensity	4	4	0.665005542102029	ns	0.204124145231932
PC4	HostRichness	4	4	0.0303828219765775	*	0.816496580927726
PC4	TotalAbundance	4	4	0.110210182673421	ns	0.6160503812913
PC4	HabitatRichness	4	4	0.0667530151696345	ns	0.707106781186547
PC4	SeasonRichness	4	4	0.429195300440349	ns	0.335410196624968
PC4	MeanIntensity	4	4	0.665005542102029	ns	0.204124145231932

The analysis revealed a strong and consistent link between niche breadth and the range of hosts utilized. Across all four ecological dimensions, generalist species exhibited significantly higher host richness than specialist species (Wilcoxon rank‐sum test, *p* = 0.03, Wilcoxon effect size *r* = 0.82 for all four axes). This indicates that a broad niche, regardless of the specific ecological dimension, is fundamentally associated with the ability to parasitize a greater number of host individuals.

In contrast, the relationship with habitat range was dimension‐specific. Significantly higher habitat richness was found only for generalists on the Seasonal Climate Gradient (PC1) (*p* = 0.018, *r* = 0.89). Niche breadth along the other three gradients (host physiology, weather exposure, and host behavior) was not significantly associated with the number of habitats occupied.

No significant differences were found between generalist and specialist groups for total abundance, seasonal richness, or mean parasitic intensity across any of the four niche dimensions (all *p* > 0.05). These results suggest that in this community, a broader niche primarily confers an advantage in the breadth of host and habitat exploitation, rather than directly translating into higher population sizes or infection intensities on individual hosts.

### 3.6 Functional Groups Defined by Multidimensional Niche Strategies

To classify the flea species into distinct functional groups based on their overall niche strategies, we performed a *k*‐means clustering analysis using their four standardized niche breadth indices as input. The analysis optimally partitioned the 12 species into four distinct functional groups, each characterized by a unique multidimensional niche profile (Table 3).

**TABLE 3:** Functional groups of flea species identified by k‐means clustering based on their multidimensional niche breadths. The table shows the number of species, the mean standardized niche breadth along each of the four principal components (PC) axes, and the species composition for each of the four identified functional groups.

Functional group	No. of species	Mean $NB_{\mathrm{PC}1}$ (climate)	Mean $NB_{\mathrm{PC}2}$ (host)	Mean $NB_{\mathrm{PC}3}$ (weather)	Mean $NB_{\mathrm{PC}4}$ (behavior)	Species included
Hyper‐generalists	2	0.992	0.916	0.994	0.945	*N. l. kuzenkoui, X. c. conformis*
Sub‐generalists	2	0.317	0.272	0.353	0.313	*O. p. praefecta, C. t. mongolicus*
Moderate specialists	7	0.159	0.145	0.165	0.160	*N. b. formis*, *X. cheopis* , *F. luculenta*, *N. l*. *ellobi*, *N. l.laeviceps*, *N. gelea*, *N. p. orientalis Loff*
Extreme specialist	1	0.000	0.000	0.000	0.000	*O. jettmari*

The first and most dominant group was termed the hyper‐generalist, consisting of the two species *N. l. kuzenkoui* and *X. c. conformis*. This group was defined by exceptionally high mean niche breadths across all four ecological gradients (mean *NB* > 0.91 for all PCs), identifying them as the unequivocal core species of the community, capable of exploiting the full range of available host‐environment conditions.

The second group, the Sub‐generalists, included *O. p. praefecta* and *C. t. mongolicus*. These species exhibited moderate niche breadths across all dimensions (mean *NB* ranging from 0.27 to 0.35), positioning them as secondary generalists that are broadly adaptable but do not possess the extreme plasticity of the hyper‐generalists.

The largest group was the Moderate Specialists, which comprised seven of the 12 species. This group was characterized by consistently low niche breadths across all four axes (mean *NB* ≈ 0.16), indicating a general strategy of specialization without being confined to an extreme degree.

Finally, the species *O. jettmari* was segregated into its own cluster, representing the extreme specialist. With a measured niche breadth of zero on all four ecological gradients, this species exemplifies the most constrained niche strategy within the community.

This classification reveals a clear, hierarchical structure of functional roles, from the dominant hyper‐generalists that define the community's niche space to a large group of moderately specialized species and finally to the highly restricted extreme specialist.

## 4 Discussion

### 4.1 Network Topology and Community Stability

Our results show that the Mongolian gerbil‐flea interaction network displays a low connectance, high nestedness, moderate modularity topology, a pattern consistent with findings from other host–parasite systems (Tylianakis et al. 2007; Poulin 2010). The significantly nested structure (NODF = 12.41), in particular, suggests a robust community architecture where specialist species tend to interact with a subset of the hosts utilized by generalists. Such a configuration is thought to enhance network stability, as the loss of peripheral or specialist species is less likely to trigger cascading extinctions (Bastolla et al. 2009; James et al. 2012).

Furthermore, the significant modularity of both the bipartite and co‐occurrence networks indicates that the flea community is partitioned into functionally cohesive subgroups. This structure likely arises from the combined influence of host heterogeneity and microenvironmental variation, as identified in our initial ordination analysis. Modularity can bolster community resilience by containing perturbations within specific modules, thus preventing system‐wide collapse (Grilli et al. 2016). Together, the combination of high nestedness and moderate modularity points to a community with inherent structural robustness.

The analysis of the co‐occurrence network further elucidated the functional roles of individual species in maintaining this structure. A clear core‐periphery organization was evident, with a small number of species acting as central hubs. Our analysis identified the hyper‐generalist, a functional group comprising *N. l. kuzenkoui* and *X. c. conformis*, as occupying the network's core, defined by their high connectivity. These core taxa functionally serve as hubs that integrate the community and likely bridge different modules (Gómez et al. 2013). In contrast, species with low connectivity, such as *O. jettmari* (the extreme specialist), were positioned at the network's periphery. Such peripheral specialists are known to be more vulnerable to environmental fluctuation and host‐population dynamics, which can lead to localized abundance declines (Krasnov, Poulin, et al. 2005). This pronounced core‐periphery structure implies that community stability is disproportionately dependent on the persistence of a few core species, whose broad resource use and high connectivity sustain the interaction patterns that underpin the entire system's robustness.

### 4.2 Seasonal Dynamics of the Parasitic Network

Seasonal network analysis indicates that the flea assemblage follows a trajectory of spring initiation, summer integration peak, autumn contraction and reorganization, a pattern that is plausibly associated with seasonal variation in temperature, humidity and host activity (Lopes et al. 2020). Extending prior studies that emphasized static topological descriptors (e.g., connectance and modularity) (Takemoto et al. 2014; Eom 2018), we explicitly incorporate seasonal environmental pressure to examine how network structure changes through the year and how such changes may reflect adaptive responses of the parasite community. This seasonal perspective broadens our understanding of parasitic‐system dynamics and helps identify key ecosystem processes and functionally important taxa. More generally, theory predicts that host‐enemy systems can shift among dynamic regimes under changing external forcing, generating nonlinear and sometimes abrupt changes in interaction structure; this provides a conceptual basis for expecting strong seasonal reconfiguration in empirical parasite networks (Kaitala et al. 1999; Yu et al. 2009).

Empirically, the summer network exhibits the highest connectance among the three seasons and a more concentrated core of taxa (nodes with degree > 75), indicating that summer corresponds to the seasonal peak in interaction strength and network integration, likely the result of elevated resource availability and favorable environmental conditions that increase encounter rates and facilitate link formation (Krasnov, Shenbrot, et al. 2005). In contrast, the autumn network shows reduced connectance and lower species richness but a significant increase in modularity, implying that the community undergoes structural reorganization through re‐modularization to cope with declining host densities and deteriorating environmental conditions (Hernandez et al. 2021). Such structural reorganization likely represents a key ecological mechanism for maintaining system stability, supporting the hypothesis that ecological networks can self‐regulate under disturbance through structural restructuring (Thébault and Fontaine 2010).

### 4.3 Multidimensional Niche Strategy and Its Functional Consequences

Our multidimensional niche analysis provides a more refined understanding of the link between a species' niche strategy and its functional role within the community. The analysis revealed a steep hierarchy of generalization, which strongly corresponded to the core‐periphery structure of the co‐occurrence network. The hyper‐generalist species (*N. l. kuzenkoui* and *X. c. conformis*), which exhibited the broadest niches across all four ecological dimensions (Figure 4), were the same species occupying the network's core (Figure 1a). Conversely, the extreme specialist (*O. jettmari*), with a niche breadth of zero on all axes, was positioned at the network's extreme periphery. This establishes a clear link: in our system, a species' centrality is not determined by its strategy on any single axis, but by its overall capacity for generalization across the entire multidimensional ecological space. This finding aligns with recent studies emphasizing that a species' functional impact is often an emergent property of multiple interacting niche axes, rather than a single trait (Poisot et al. 2012; Carmona et al. 2021).

Importantly, our analysis reveals a critical distinction: a broader niche does not automatically translate into higher local abundance or parasitic intensity. Our quantitative comparisons showed that while generalists exploited a significantly wider range of hosts and habitats (Table 2, Figure 4), we found no significant difference in either their total abundance or mean parasitic intensity compared to specialists across any of the four ecological dimensions (Wilcoxon rank‐sum test, all *p* > 0.05).

This key finding suggests that the primary advantage of a generalist strategy in this system is an expansion of scope, not an increase in local intensity. This resonates with the ongoing debate on the relationship between niche breadth and species abundance, where recent large‐scale analyses have shown this link to be highly context‐dependent and often non‐linear (Saupe et al. 2015). Generalists appear to follow a “bet‐hedging” strategy of broad exploitation but low density on any given host individual or in any single location. This empirically supports a classical ecological trade‐off, where generalization across habitats or resources does not always confer dominance in terms of local population size, a mechanism that can facilitate the coexistence of both generalists and specialists (Levins 1968).

### 4.4 Functional Groups and Mechanisms of Species Coexistence

While traditional analyses often focus on pairwise niche overlap as a proxy for competition (Schoener 1974), our multidimensional niche framework allows for a more holistic view of the community's coexistence mechanisms. By classifying species into functional groups based on their complete niche profiles, we propose that coexistence in this flea community is maintained not just by fine‐scale resource partitioning, but by the adoption of fundamentally different large‐scale ecological strategies. This approach, which defines functional groups based on multidimensional trait space, is increasingly recognized as a powerful way to predict community assembly and ecosystem functioning (Pigot et al. 2018; Rudolf 2019).

Our analysis identified a clear hierarchy of four distinct functional groups. The hyper‐generalist (*N. l. kuzenkoui, X. c. conformis*) represents the community's dominant strategists. Their ability to occupy nearly the entire available niche space suggests they likely function as “core species” that shape resource availability and influence the entire community structure (Bitam et al. 2010; Khokhlova et al. 2012). The coexistence of two hyper‐generalists, rather than competitive exclusion, implies that even at this broad scale, some form of limiting similarity or subtle partitioning must be at play. Recent theory suggests that such coexistence among highly similar dominant species can be stabilized by subtle temporal niche partitioning or priority effects, which are difficult to detect in time‐averaged data (Fukami 2015). Furthermore, their dominance is likely actively maintained through the configuration of their competitive interactions and resource‐occupation patterns, a mechanism recently proposed to stabilize the position of core species across heterogeneous conditions (Krasnov et al. 2024).

The persistence of a large group of moderate specialists (seven species) alongside these dominant generalists is a key feature of this community's structure. These species likely survive through a combination of mechanisms. They may avoid direct competition by utilizing resource peripheries not efficiently exploited by the generalists, or engage in fine‐scale partitioning of host characteristics or microhabitats that our broad‐scale analysis did not capture (Krasnov et al. 2006). The existence of an extreme specialist (*O. jettmari*) further reinforces this, suggesting the presence of highly stable, predictable micro‐niches that can be exclusively exploited.

This partitioning of the community into distinct strategic groups—from dominant, all‐encompassing generalists to highly restricted specialists—provides a clear mechanism for stable coexistence. It moves beyond the question of simple resource overlap to a more dynamic view of community assembly, where different combinations of adaptive traits allow species to succeed in different ways. This aligns with modern coexistence theory, which posits that stability arises from the various ways species respond to and influence their environment, a concept known as the “multidimensionality of the niche” (Chesson 2000). Indeed, empirical evidence for high‐dimensional niche differentiation as a key driver of diversity in complex communities is rapidly accumulating (Levine et al. 2017).

While our study provides high‐resolution insights into the hierarchical organization of flea communities within the fundamental unit of a dominant host ( *M. unguiculatus* ), future investigations extending this framework to multi‐host systems and longer temporal scales would be valuable. Such expansion would help determine whether this hierarchical structure persists under more complex cross‐species transmission pathways and inter‐annual climate oscillations. Furthermore, the hypothesized bet‐hedging strategy driving the dominance of hyper‐generalists offers a compelling avenue for further research, where experimental manipulation or functional trait analysis could fully elucidate the mechanisms facilitating their coexistence with specialists.

## 5 Conclusion

This study provides a novel, multidimensional perspective on the niche structure and assembly rules of a single‐host flea community. Our results show that the community is not static, but exhibits profound seasonal dynamics in its network structure, adapting its connectivity and modularity to fluctuating environmental conditions. By integrating this dynamic network perspective with a quantitative, multi‐axis niche framework, we moved beyond the traditional generalist‐specialist dichotomy to reveal a more complex and hierarchical community organization.

Our central finding is that, within this seasonally dynamic Mongolian gerbil‐flea system, the community is internally structured by a steep hierarchy of generalization. A small number of hyper‐generalist species occupy the core of the ecological and network space across all seasons, a role defined by their broad niche across multiple ecological dimensions (seasonal climate, host physiology, weather, and host behavior). Furthermore, we found that a broader niche primarily confers an advantage in the scope of resource exploitation (host and habitat richness) rather than in local dominance (abundance or parasitic intensity), highlighting a key ecological trade‐off that likely facilitates species coexistence within this structured system.

In conclusion, our work establishes a robust framework for dissecting the interplay between species' niche strategies, network dynamics, and community structure in parasitic systems. These findings not only provide a deeper mechanistic understanding of stability in the Mongolian gerbil‐flea system but also offer a powerful analytical pathway for future research into how such communities will respond to ongoing environmental change.

## Author Contributions

**Rui Geng:** conceptualization (equal), methodology (equal), validation (equal), writing – review and editing (equal). **Yakun Liu:** conceptualization (equal), methodology (equal), validation (equal), writing – review and editing (equal). **Haizhou Yang:** data curation (equal), investigation (equal), validation (equal). **Guokang Chen:** data curation (equal), investigation (equal), validation (equal). **Shuai Yuan:** funding acquisition (equal), methodology (equal), project administration (equal), resources (equal), supervision (equal), writing – review and editing (equal). **Heping Fu:** funding acquisition (equal), project administration (equal), resources (equal), supervision (equal), validation (equal), writing – review and editing (equal).

## Funding

This work was supported by National Key Research and Development Program of China, 2024YFD1400501, 2024YFD1400503, 2024YFD1400505. The Major Science and Technology Project of Inner Mongolia Autonomous Region, 2021ZD0006 specialised Projects for Scientific Research in First‐Class Disciplines, YLXKZX‐NND‐005, YLXKZX‐NND‐031. The Basic Scientific Research Business Expenses of Universities Directly Under Inner Mongolia Autonomous Region, BR221307, BR220106, BR221037. The National Natural Science Foundation of China, 32060256, 32060395. The Grassland Ecological Protection and Restoration Treatment Subsidy, RK2200000355. The Inner Mongolia Natural Science Foundation, 2023MS03025. The Inner Mongolia Autonomous Region Science and Technology Program, 2021GG0108. The 2022 Inner Mongolia Autonomous Region Youth Science and Technology Talent Development Plan, NJYT22044.

## Conflicts of Interest

The authors declare no conflicts of interest.

## Data Availability

The datasets and code generated and/or analyzed during the current study are openly available in the Zenodo repository at https://doi.org/10.5281/zenodo.18074994.
